# Comparisons of outcomes and complications of immediate sequential bilateral cataract surgery and unilateral cataract surgery in a tertiary hospital in South Korea

**DOI:** 10.1038/s41598-022-26851-2

**Published:** 2022-12-26

**Authors:** Suji Hong, Wonkyung Park, Youngsub Eom, Hyo Myung Kim, Jong Suk Song

**Affiliations:** 1 Department of Ophthalmology, Korea University Guro Hospital, 148, Gurodong-ro, Guro-gu, Seoul, Republic of Korea; 2 Department of Ophthalmology, Korea University Anam Hospital, 73, Koreadae-ro, Seongbuk-gu, Seoul, Republic of Korea; 3 Department of Ophthalmology, Korea University Ansan Hospital, 123, Jeokgeum-ro, Danwon-gu, Ansan-si, Gyeonggi-do Republic of Korea

**Keywords:** Diseases, Medical research

## Abstract

We investigated the proportions of immediate sequential bilateral cataract surgery (ISBCS) and unilateral cataract surgery during the coronavirus disease 2019 pandemic and compared visual outcomes between the two groups in a tertiary hospital in South Korea. We reviewed 441 cataract surgeries performed between March 1, 2021, and October 31, 2021, at Korea University Guro Hospital by a single surgeon (J.S.S). Medical records of demographics, preoperative visual acuity, corneal astigmatism, axial length, preoperative spherical equivalent, preoperative target (using Barrett’s Universal 2 formula), postoperative visual acuity, postoperative refractive error, and postoperative complications were evaluated. Among all patients, 322 (73.0%) eyes underwent ISBCS, and 119 (27.0%) eyes underwent unilateral cataract surgery. The preoperative corrective distance visual acuity (CDVA) was lower in the unilateral cataract surgery group (0.40 ± 0.45 logMAR) than the ISBCS group (0.28 ± 0.16 logMAR, *P* = 0.008), whereas there was no significant difference in postoperative CDVA between the two groups (0.06 ± 0.10 logMAR vs. 0.07 ± 0.16 logMAR, *P* = 0.63). There was also no difference in the absolute refractive error between the two groups (0.46 ± 0.37 diopters [D] vs. 0.42 ± 0.38 D, *P* = 0.63). The preoperative CDVA (*P* = 0.000) was the significant factor influencing absolute refractive error (r = 0.191, *P* < 0.001). There was no difference in complications between the two groups, although two patients in the ISBCS group complained of postoperative strabismus.

## Introduction

Cataract surgery is one of the most commonly performed surgeries worldwide. Accordingly, many researchers have tried to find time and cost-efficient methods for cataract surgery. Immediate sequential bilateral cataract surgery (ISBCS) was first suggested in 1952^1^. However, after 70 years, delayed sequential bilateral cataract surgery (DSBCS) is still the standard for bilateral cataract surgery in most countries^2,3^. In 2015, the American Society of Cataract and Refractive Surgeons reported that about 70% of surgeons worldwide and 80% of surgeons in the United States answered that they would perform DSBCS rather than ISBCS^4^. The preference might have been influenced by the controversies about bilateral postoperative complications and predictability of postoperative refractive errors^5,6^. Also, the insurance system may contribute to low preference of ISBCS in some countries. For instance, in the United States, where the Medicare system only pays for 50% of the reimbursement for the second operation in ISBCS patients, only 0.20% of all cataract surgeries was ISBCS between 2011 and 2019^7^.

However, during the coronavirus disease 2019 (COVID-19) pandemic, interest in immediate sequential bilateral cataract surgery (ISBCS) increased^8,9^ because the pandemic increased the need to minimize face-to-face contact, and ISBCS requires fewer follow-up visits and re-examinations. ISBCS also has advantages in terms of facilitating efficient use of operating rooms, enabling more surgeries to be scheduled^10,11^. It is also beneficial for the patient as the total recovery time and associated costs are reduced^5,12,13^.

In South Korea, ISBCS could be an advantageous strategy for disease severity management in tertiary hospitals. According to the criteria of the Korea Health Insurance Review and Assessment service, minor diseases (which are designated as disease group C) must less than 8.4% of the total diseases treated in tertiary hospitals. As unilateral cataract surgeries belong to disease group C, only a limited number of cataract surgeries are allowed in tertiary hospitals. However, because bilateral cataract is categorized as a moderate disease (disease group B), tertiary hospitals do not need to reduce the number of surgeries if cataract operations on both eyes are scheduled for the same day.

Although many studies have recently discussed the importance of ISBCS during the COVID-19 pandemic and suggested new protocols to be implemented during the pandemic period^14^, no studies have investigated the proportion of ISBCS procedures performed in tertiary hospitals in Asia. Thus, this study aimed to investigate the proportions of patients who underwent unilateral cataract surgery and ISBCS in a tertiary hospital in South Korea. In addition, we also compared visual outcomes and postoperative complications between the two patient groups.

## Methods

### Patient selection

This study was conducted after obtaining approval from the Institutional Review Board of the Guro Hospital of Korea University and adhered to the Declaration of Helsinki. We retrospectively reviewed the clinical records of patients who underwent cataract surgery between March 1, 2021, and December 31, 2021, at Guro Hospital of Korea University performed by a single surgeon (J.S.S). Informed consent was obtained from all subjects.

Patients were recommended to receive ISBCS if they were indicated to undergo cataract surgery on both eyes. If patients refused ISBCS, then the surgeries were scheduled separately more than one week apart. According to surgery schedule, patients were divided into two subgroups. The ISBCS group included patients who underwent two cataract operations on the same day. The unilateral cataract surgery group included patients who had cataract surgery performed on only one eye or patients who had two cataract operations performed more than one week apart.

Medical records, including age, laterality, preoperative corrective distance visual acuity (CDVA) (logMAR), corneal astigmatism (D), axial length (mm) as measured with the IOLMaster 500 (Carl Zeiss Meditec AG, Jena, Germany), and preoperative spherical equivalent (SE) (D), were evaluated for all patients. Also, the preoperative target calculated using Barrett’s Universal 2 formula was included in the preoperative data.

Four types of intraocular lens(IOLs) were implanted according to patient preference and considering the cost and functions of each, including a monofocal IOL (TECNIS 1-piece ZCB00 IOL; Johnson & Johnson Vision Care, Inc., Jacksonville, FL, USA), extended depth-of-focus(EDOF) IOL (TECNIS Eyhance ICB00; Johnson & Johnson Vision Care, Inc.), multifocal IOL (TECNIS Multifocal ZMB00; Johnson & Johnson Vision Care, Inc.), and toric IOL (TECNIS Toric ZCT; Johnson & Johnson Vision Care, Inc.).

Patients with corneal opacity, retinal disorders, glaucoma, myopic correction >  − 1.5 D, or whose axial length could not be measured with the IOL Master 500 system were excluded.

When patients visited the clinic one month after their final surgery, postoperative CDVA and postoperative refractive error (RE) (postoperative SE−IOL target D value) were compared between the two groups. Postoperative complications, including postoperative endophthalmitis, prolonged corneal edema, cystoid macular edema(CME), and postoperative strabismus, were also evaluated one month after surgery.

### Surgical methods

All surgeries were performed by a single surgeon (J.S.S) under topical anesthesia of 4% lidocaine and 0.5% proparacaine hydrochloride (Alcaine [Alcon Laboratories Inc., Fort Worth, TX, USA] or Paracaine [Hanmi Pharm, Seoul, Korea]). After making a 2.20-mm clear corneal incision, forceps were used to create a continuous curvilinear capsulorrhexis. Phacoemulsification was performed via a stop and chop technique. Then, the folded IOL was inserted into the capsular bag through the corneal incision.

With ISBCS patients, each operation was performed as a separate procedure, with total segregation of the two surgeries. After operating on the first eye, the operators, including the surgeon and assistants, changed gowns and gloves. Then, the second surgery was conducted, adopting the same steps as those followed for the first eye. Also, to prevent incorrect switching of the IOLs, before starting each surgery the second and first assistants checked the patient's data and IOL information. Then, the surgeon (J.S.S) checked the patients’ information including their name, laterality and power of the IOL.All patients were treated with topical 0.5% levofloxacin (Cravit; Santen Pharmaceutical, Osaka, Japan) and 0.1% bromfenac sodium (Bronuck; Taejoon Pharm, Seoul, Korea) twice daily and with a topical steroid eyedrop (0.1% fluorometholone; Santen Pharmaceutical) four times daily from three days before cataract surgery to four weeks after cataract surgery.

### Data analysis

The primary result was the proportion of surgeries performed in a tertiary hospital. The preoperative data, including age, CDVA, corneal astigmatism, SE, axial length, and IOL power, of the patient groups were compared using Student’s *t* test. Student’s *t* test was also used to assess the differences in postoperative CDVA and postoperative RE between the two groups. Pearson’s chi-square test was used to compare the distribution of IOL types between the two groups. Simple linear regression analysis was performed to assess the correlation between the preoperative data and postoperative RE. The frequencies of postoperative complications in the two groups were compared using Fisher’s exact test. Statistical analysis was performed using IBM SPSS for Windows version 21.0 (IBM Corporation, Armonk, NY, USA), and a *P* < 0.05 was considered to be statistically significant.

## Results

### Patient demography and intraocular lens (IOL) distribution

**Figure 1:**
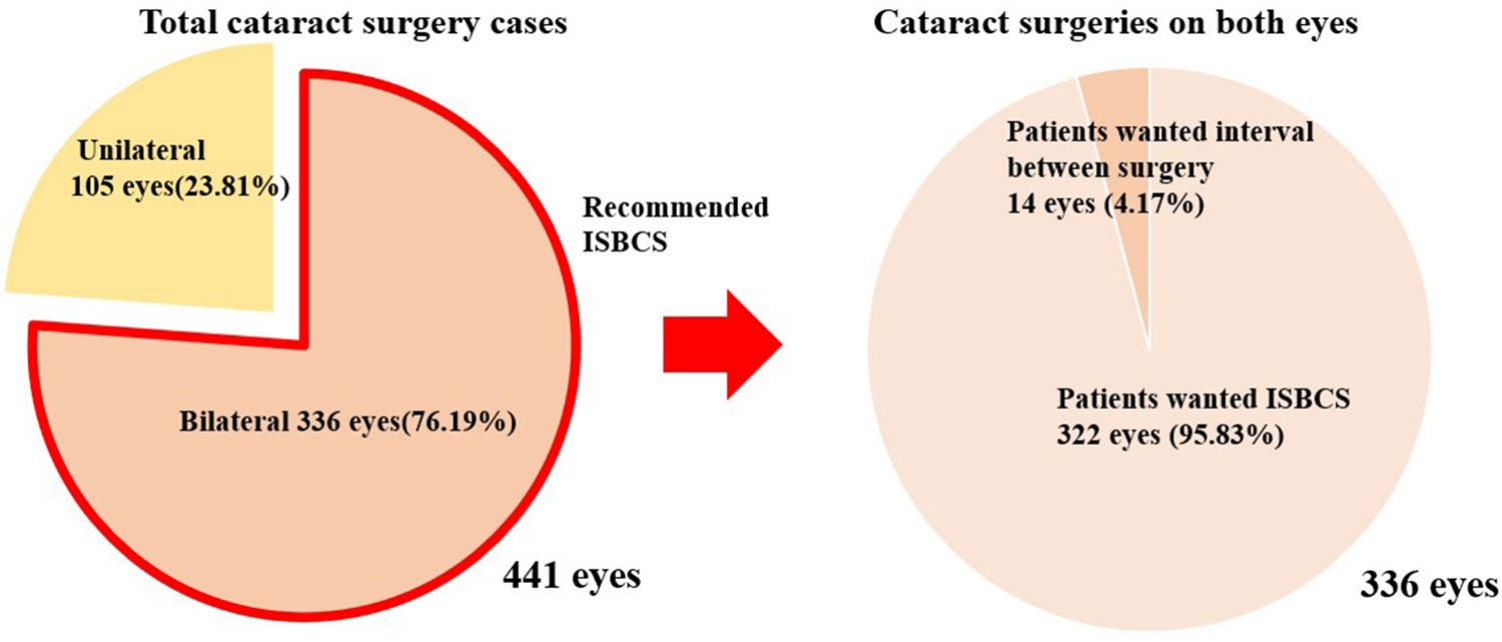
Distribution of cataract surgery patients. ISBCS, immediate sequential bilateral cataract surgery.

We reviewed 441 cases of cataract surgeries at Guro hospital from March 1, 2021, to December 31, 2021. Among a total of 441 cases, 336 eyes of 168 patients were indicated for cataract surgery on both eyes and 105 eyes of 105 patients were indicated for cataract surgery on one eye. When bilateral cataract patients were recommended to undergo ISBCS, 322 eyes (95.83%) underwent surgeries on the same day. Fourteen eyes (4.17%) refused same-day surgery and decided to maintain an interval between surgeries more than 1 week (Fig. 1).

Among the total of 441 eyes operated on, 338 were divided into two groups. The unilateral cataract surgery group included 77 patients (22.78%) and the ISBCS group included 261 patients (77.22%). There was no difference in corneal astigmatism (K2–K1) (D), preoperative SE (D), axial length (mm), or IOL power (D) between the two groups (*P* = 0.052, *P* = 0.681, *P* = 0.883, *P* = 0.831, and *P* = 0.993, respectively). The CDVA was lower in the unilateral cataract surgery group (0.40 ± 0.45 logMAR) than the ISBCS group (0.28 ± 0.30 logMAR, *P* = 0.008). The average age of the patients in the ISBCS group tended to be higher, although the difference was not statistically significant (69.2 ± 9.40 vs. 66.65 ± 11.26 years, *P* = 0.052) (Table 1).

**Table 1:** Patient demographics and preoperative data.

	Group 1 Unilateral cataract surgery (n = 77)	Group 2 ISBCS (n = 261)	*P* value
Age (years)	66.65 ± 11.26	69.15 ± 9.40	0.052*
Preoperative CDVA (logMAR)	0.40 ± 0.45	0.28 ± 0.30	0.008*
Corneal astigmatism (K2-K1)	0.84 ± 0.82	0.90 ± 1.27	0.681*
Preoperative SE (D)	0.05 ± 2.18	0.09 ± 2.60	0.883*
Axial length (mm)	23.64 ± 0.89	23.60 ± 1.1.8	0.831*
IOL power (D)	21.17 ± 2.33	21.20 ± 3.37	0.993*
**IOL distribution**			
Monofocal IOL	55 (71.43%)	181 (69.35%)	
Multifocal IOL	11 (14.29%)	38 (14.56%)	
EDOF IOL	9 (11.69%)	40 (15.33%)	
Toric IOL	2 (2.60%)	2 (0.77%)	
Total	77 (100%)	261 (100%)	0.519^†^

CDVA, corrected distance visual acuity; EDOF, extended depth-of-focus; IOL, intraocular lens; ISBCS, immediate sequential bilateral cataract surgery; SE, manifest spherical equivalent.

*Student’s *t* test.

^†^Pearson’s chi-square test.

Patients with monofocal IOLs made up the largest proportion in each group, accounting for 71.43% of the unilateral group and 69.53% of the ISBCS group. In the unilateral group, patients with multifocal IOL accounted for the second-largest proportion (14.29%), followed by those with EDOF IOLs (11.69%) and toric IOLs (2.60%). In the ISBCS group, EDOF IOLs were the second-most common IOL (15.33%), followed by multifocal IOLs (14.56%) and toric IOLs (0.77%). There was no difference in the distribution of IOLs between the two groups (*P* = 0.519) (Table 1).

### Visual outcome and postoperative complications

One month after surgery, the postoperative CDVA was 0.06 ± 0.10 logMAR in the unilateral group and 0.07 ± 0.16 logMAR in the ISBCS group, and this difference was not statistically significant (*P* = 0.63). There was also no statistically significant difference in postoperative SE (− 0.24 ± 0.62 diopters [D] vs. 0.19 ± 0.59 D, *P* = 0.625). The postoperative absolute RE was also not statistically different between the two groups (0.46 ± 0.37 D vs. 0.42 ± 0.38 D, *P* = 0.482) (Table 2).

**Table 2:** Clinical outcomes one month after surgery.

	Group 1 Unilateral cataract surgery (n = 77)	Group 2 ISBCS (n = 261)	*P* value
Preoperative target (D)	− 0.33 ± 0.20	− 0.44 ± 0.15	0.487*
Postoperative CDVA (logMAR)	0.06 ± 0.10	0.07 ± 0.16	0.63*
Postoperative SE (D)	− 0.24 ± 0.62	0.19 ± 0.59	0.625*
Absolute value of postoperative RE (D)	0.46 ± 0.37	0.42 ± 0.38	0.482*

CDVA, corrected distance visual acuity; ISBCS, immediate sequential bilateral cataract surgery; RE, refractive error; SE, manifest spherical equivalent.

*Student’s *t* test.

Refractive error (postoperative spherical equivalent − intraocular lens target in diopters).

Eyes with a postoperative RE within ± 0.5 D accounted for 64.94% of the unilateral group and 72.41% of the ISBCS group. The postoperative RE was within ± 1.00 D in 93.51% of the unilateral group and in 96.17% of the ISBCS group. There was no significant difference between the two groups (*P* = 0.205 and *P* = 0.319) (Table 3).

**Table 3:** Distribution of patients with postoperative refractive error in the two groups.

	Group 1 Unilateral cataract surgery (n = 77)	Group 2 ISBCS (n = 261)	*P* value
− 0.5 < postoperative RE^‡^ < 0.5	50 (64.94%)	189 (72.41%)	0.205*
− 1.0 < postoperative RE < 1.0	72 (93.51%)	251 (96.17%)	0.319*

ISBCS, immediate sequential bilateral cataract surgery; RE, refractive error.

*Pearson’s Chi-square test.

^‡^Refractive error (postoperative spherical equivalent − intraocular lens target in diopters).

**Figure 2:**
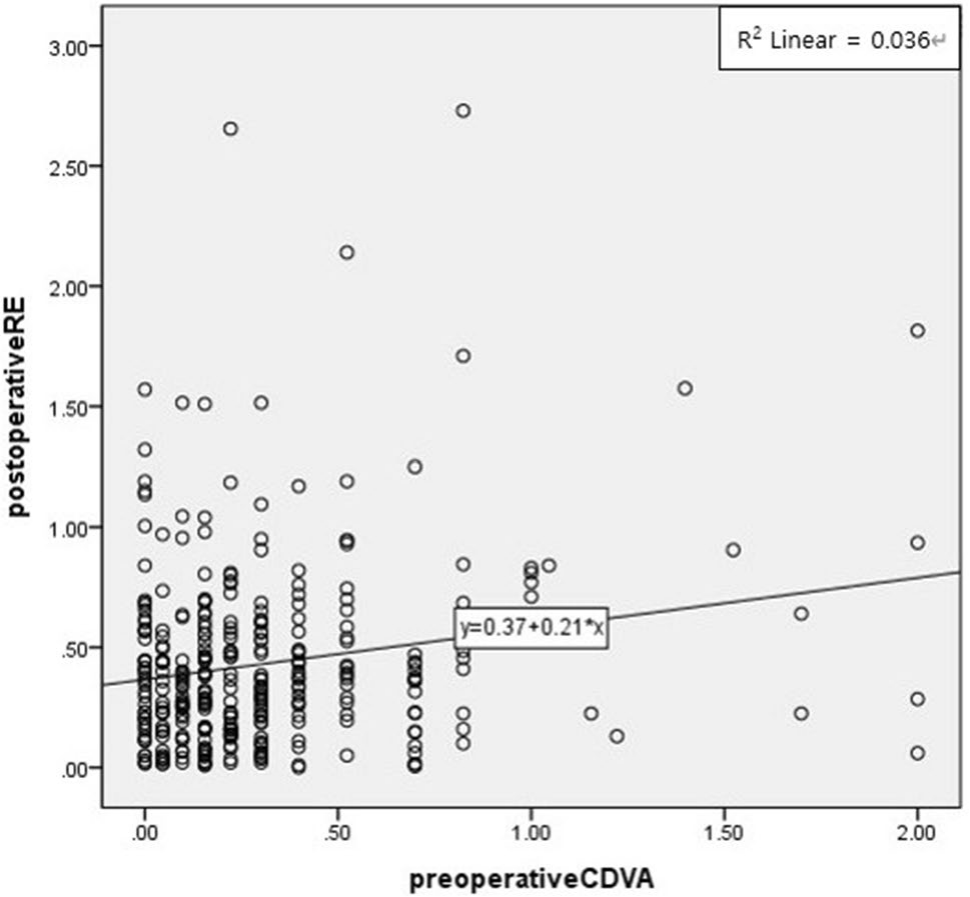
Relationship between corrected distance visual acuity and refractive error. RE, refractive error.

In the linear regression analysis performed to find factors affecting postoperative absolute RE, only preoperative CDVA (*P* < 0.001) was a significant factor among the preoperative data. The equation for the regression line was postoperative RE =  + 0.37 + 0.21 × preoperative CDVA (logMAR) (R^2^ = 0.036) (Table 4 and Fig. 2). In the multivariate regression analysis, preoperative CDVA and axial length correlated with postoperative absolute RE (*P* < 0.001 and *P* = 0.025) (Table 5).

**Table 4:** Linear regression analysis of postoperative absolute refractive error and preoperative data.

Preoperative data	Unstandardized coefficients	Standardized coefficients	*P* value
Preoperative CDVA (logMAR)	0.211	0.191	< 0.001*
Corneal astigmatism (K2–K1)	− 0.007	− 0.21	0.702*
Preoperative SE (D)	− 0.009	− 0.043	0.430*
Axial length (mm)	− 0.020	− 0.058	0.285*

CDVA, corrected distance visual acuity; SE, manifest spherical equivalent.

*Simple linear regression analysis.

**Table 5:** Multivariate regression analysis of postoperative absolute refractive error and preoperative data.

Preoperative data	Partial regression coefficients	Standardized partial regression coefficients	*P* value
Preoperative CDVA (logMAR)	0.225	0.202	< 0.001*
Corneal astigmatism (K2–K1)	− 0.020	− 0.062	0.260*
Preoperative SE (D)	0.001	0.005	0.939*
Axial length (mm)	− 0.051	− 0.150	0.025*

CDVA, corrected distance visual acuity; SE, manifest spherical equivalent.

*Multivariate regression analysis.

Endophthalmitis was not observed in the two groups. CME was diagnosed in one eye in the unilateral group (0.01%) and two eyes in the ISBCS group (0.01%), with no difference in frequency between the two groups (*P* = 0.541). There was no difference in the frequency of prolonged corneal edema between the 2 groups (2 eyes [0.03%] vs. 5 eyes [0.02%], *P* = 0.660). Two patients in the ISBCS group complained of postoperative strabismus, with no statistically significant difference between the groups (0 eyes vs. 2 eyes [0.77%], *P* = 0.274) (Table 6). One patient had intermittent exotropia, and another had right hypertropia.

**Table 6:** Incidence of postoperative complications one month after surgery.

	Group 1 Unilateral cataract surgery (n = 77)	Group 2 ISBCS (n = 261)	*P* value
Postoperative cornea edema	2	5	0.660*
Postoperative strabismus	0	4	0.274*
Postoperative endophthalmitis	0	0	–
Exudative inflammatory response	1	2	0.541*
CME	0	2	1.00*

CME, cystoid macular edema; ISBCS, immediate sequential bilateral cataract surgery.

*Fisher’s exact test.

## Discussion

The rate of ISBCS varies from country to country depending on the health care system or insurance system^7,15,16^. Finland has the highest rate of ISBCS, accounting for about 60% of all cataract surgeries. In contrast, the rate of ISBCS is very low in the United States (0.2%), possibly because the Medicare system only pays 50% of the reimbursement for the second operation if both cataract operations are performed on the same day^7^. In this study, 73% of cataract surgeries were ISBCS procedures, and if patients were recommended to undergo bilateral cataract surgery, 96% of patients decided to receive both operations on the same day. Even though this study was limited to procedures performed by a single surgeon, the results suggest a high preference for bilateral surgeries on the same day among patients during the COVID-19 pandemic in South Korea.

The average age of the ISBCS group tended to be higher, though the difference was not statistically significant, and this is consistent with the results of previous studies suggesting that older people or people with reduced mobility prefer ISBCS^7,17^. The preoperative CDVA (logMAR) was lower in the unilateral group. There is a possibility that the unilateral group had more advanced cataracts. However, we also assume that patients' concerns might have had an effect in making such a decision. Likewise, Alwankar et al. reported that patients with ocular comorbidities were less likely to undergo ISBCS^18^.

One of the major controversies about ISBCS is the predictability of target refraction. There are some concerns that there is no chance to plan the second surgery on the basis of the results of the first eye^5,6,17,19^. Jibrajka et al. found that there was significant improvement in the predicted RE of the second surgery when the first surgery result was reflected^6^. On the other hand, Kessel et al. reported that none of the existing randomized controlled clinical trials could provide evidence of the prevalence of postoperative anisometropia in patients undergoing ISBCS^20^. In this study, there was no difference in postoperative CDVA or postoperative RE between the bilateral group and the unilateral group.

During simple linear regression analysis, preoperative CDVA showed a correlation with postoperative RE, as in previous studies^21,22^. This indicates that lower preoperative CDVA leads to a higher absolute RE because it impacts the preoperative exam. In the multivariate regression analysis, it was determined that axial length also affected the RE. These analyses suggest that postoperative factors rather than whether the patient underwent ISBCS or not determine how postoperative absolute RE is affected by axial length or CDVA. Also, 72.41% of ISBCS patients achieved a postoperative RE within ± 0.5 D, and 96.17% of ISBCS patients achieved a postoperative RE within ± 1.0 D, which is a substantially improved result compared to those reported by Johansson et al. or Guber et al.^23–25^. We believe this improvement is mainly attributable to using the Barret Universal 2 formula, which has been found to facilitate better predictability compared to other formulae^26^. Further improvement of the formula used as well as the higher accuracy of optical biometry and tomography could reduce dependence on the outcome of the first surgery.

Another important objection against ISBCS is the risk of bilateral vision loss as a result of bilateral complications^27^. Although there was no endophthalmitis patient in this study, endophthalmitis is the most feared “catastrophic” complication that could lead to vision loss. However, the frequency of endophthalmitis in ISBCS is similar to that observed during unilateral cataract surgery, occurring in 0.02–0.05% of cases. In addition, there were no reported cases of bilateral endophthalmitis after ISBCS when appropriate guidelines were followed^16,28–30^. If the sequential surgeries are performed independently with strict hygienic precautions, including re-draping, re-scrubbing of lids, and using separate surgical devices after the first surgery, the risk of bilateral endophthalmitis could be reduced^20,31^.

There was no difference in the frequencies of other postoperative complications. However, two patients in the ISBCS group complained of postoperative strabismus.

One patient had a history of strabismus surgery and the other patient was diagnosed right hypertropia due to superior oblique palsy. Previous studies reported that decompensated phoria in long standing SO palsy frequently produces diplopia after cataract surgery^32–34^. Consequently the cause of postoperative strabismus for both patients was decompensation of pre-existing strabismus.

Strabismus can occur after cataract surgery and refractive surgery, and Gunton et al. found that, following cataract surgery with topical anesthesia, decompensation of pre-existing strabismus is the leading cause of postoperative strabismus^35^. No studies have assessed the relationship between postoperative strabismus and ISBCS. However, we suggest that the dramatic change in binocular RE in the early postoperative period might disrupt binocular fusion. It seems that further study is needed in order to evaluate the risk of postoperative strabismus following ISBCS. Surgeons need to be aware of the possibility of developing strabismus after ISBCS. Strabismus history-taking would also be helpful.

A previous study by Carolan et al. reported that patients prefer receiving surgeries on the same day over having surgeries with ≥ 4 weeks interval between two surgeries with a decreased possibility (5%) of wearing glasses^36^. The result shows that patients consider convenience to be an important factor in their decision alongside reductions in postoperative RE.

We suggest that ISBCS needs to be more widely considered, taking patient’s preferences into account. Especially in Korea, cataract surgery falls under the diagnosis-related group payment system in which payments are fixed, and the operation fee for the first operation is the same for the second one. ISBCS can be an advantageous operative strategy for tertiary hospitals, not only in order to meet the criteria of the Korea Health Insurance Review and Assessment Service, but also to improve convenience for patients.

There are some limitations in this study. First, this study was limited to procedures performed by a single surgeon (J.S.S) during a relatively short period of time. Second, the visual outcome and postoperative complications were evaluated one month after surgery. Also, this study did not collect data on patients’ satisfaction after ISBCS.

In conclusion, results show that the majority of East Asian patients in South Korea willingly consented to ISBCS: specifically, when patients were recommended to undergo ISBCS, 95.83% of patients consented. There was no significant difference in visual outcomes or complications between the ISBCS and unilateral cataract surgery groups. However, postoperative diplopia was observed only with ISBCS group patients and decompensation of pre-existing strabismus was the cause.

## Data Availability

The raw data for this study are available upon reasonable request from the corresponding author.
